# Influence of alcoholism and gender on the relationship between personality and drinking motivation

**DOI:** 10.1186/1940-0640-10-S1-A56

**Published:** 2015-02-20

**Authors:** Susan Mosher Ruiz, Mary M Valmas, Kayle S Sawyer, Maaria I Kemppainen, Marlene Oscar-Berman

**Affiliations:** 1 Department of Anatomy & Neurobiology, Boston University School of Medicine, Boston, MA, 02118, USA; 2 Psychology Research Service, VA Boston Healthcare System, Jamaica Plain, MA, 02130; 3 Departments of Psychiatry and Neurology, Boston University School of Medicine, Boston, MA, 02118, USA

## Background

Alcoholic men and women tend to have differential patterns of associated comorbid psychiatric disorders, distinct cognitive and emotional abnormalities, and varying corresponding structural and functional brain abnormalities. Further, although converging, there remain gender differences in sociocultural norms related to alcohol use behaviors. As such, men and women may be motivated to use and abuse alcohol for different reasons. Previous literature has suggested a role for personality in drinking motives, but this relationship generally has been assessed as a risk factor for adolescents and young adults rather than in an adult population. The goal of the present study was to determine how alcoholism and gender are related to the associations between personality traits and drinking motivation.

## Methods

Participants included 67 abstinent long-term alcoholic adults (31 women) and 66 age-equivalent nonalcoholic controls (31 women). Personality characteristics were assessed with the 57-item Eysenck Personality Questionnaire-Revised Short Scale, which includes extraversion, neuroticism, psychoticism, and lie scales. To evaluate drinking motivation, participants completed the 20-item self-report Drinking Motives Questionnaire Revised (DMQ-R), an instrument developed for use in adolescents that recently has been validated for adults. The DMQ-R assesses frequency of motives for drinking alcohol falling into the categories of enhancement, social, conformity, and coping.

## Results

Both alcoholic men and women scored higher than their respective same-sex controls on the personality trait of neuroticism, while controls of both genders scored higher than alcoholics on the lie scale. Alcoholic women additionally scored higher than control women on psychoticism. Women scored higher than men on neuroticism, particularly among alcoholics, and men scored higher than women on psychoticism, particularly among controls. With respect to the measures of motivations for drinking, alcoholics of both genders scored higher than their respective controls on all four drinking motivation factors. For both alcoholic men and women, mean scores for enhancement and coping were highest, followed closely by social factors, with conformity indicated least often. Nonalcoholic control men and women scored highest for social and enhancement factors, with coping and conformity indicated less frequently. While significant gender differences in drinking motivation were not observed for either alcoholics or controls, there were differences in the relationships between personality and drinking motivation. Alcoholic women displayed inverse relationships between extraversion and the conformity drinking motivation factor, and between lie scores and coping. Moreover, alcoholic women showed a positive relationship between both neuroticism and psychoticism scores and coping. Among alcoholic men, lie scores were inversely associated with enhancement, coping, and conformity motivation ratings. Control men displayed a positive relationship between neuroticism and conformity scores, and control women showed a negative relationship between lie and enhancement scores.

## Conclusions

The relationships between personality and drinking motivation vary with alcoholism and gender. Compared to same-sex controls, personality traits of alcoholics reflected higher psychopathology and stronger motives for drinking. Additionally, alcoholic men and women differed with respect to the relationships between personality traits and motives for drinking. A better understanding of how different personality traits affect drinking motivations for alcoholic men and women can inform individualized relapse prevention strategies.

